# Hypothetical impact of the Mexican front-of-pack labeling on intake of critical nutrients and energy

**DOI:** 10.1186/s41043-023-00462-7

**Published:** 2023-11-08

**Authors:** Paola Villaverde, Lizbeth Tolentino-Mayo, Carlos Cruz-Casarrubias, Juan C. Salgado, Tania C. Aburto, Simón Barquera

**Affiliations:** 1 Center for Nutrition and Health Research, National Institute of Public Health, Avenida Universidad 655, Santa María Ahuacatitlán, 62100 Cuernavaca, Morelos Mexico; 2 National Council of Humanities, Science and Technology, Mexico. Av. de los Insurgentes Sur 1582, Crédito Constructor, Benito Juárez, 03940 Mexico City, CDMX Mexico

**Keywords:** Front-of-pack warning labels (FOPWL), Official Mexican Standards (NOM-51), Processed food, Nutrition, Energy, Saturated fat, Trans-fat, Added sugars, And sodium

## Abstract

**Background:**

Since 2020 in Mexico, front-of-pack warning labels (FOPWL) have been implemented in processed products. Evidence supports warning labels allow consumers to identify unhealthy products. We aimed to evaluate the hypothetical impact of the FOPWL regulation on the Mexican population’s intake of critical nutrients and energy, based on the replacement of food and beverages using 4 hypothetical scenarios which represented the phases of the regulation.

**Methods:**

Dietary data were collected using a standardized 24-h dietary recall from the 2016 Mexican National Health and Nutrition Survey (*n* = 4184). To evaluate the hypothetical impact of FOPWL, the nutritional content of critical nutrients in processed products was evaluated according to the Mexican Official Standard 051 (NOM-051). Then, we replaced products with several warning labels (WL) with those with a fewer number of them or with non-processed food. For the replacement, 4 hypothetical scenarios were established: scenario 1: the current consumption of the Mexican Population, scenario 2: the replacement during the first phase of the norm, scenario 3: the replacement in the second phase and scenario 4: the replacement in the last phase. We estimated the means, confidence intervals (CI 95%), and the mean percentage change of energy, saturated fat, trans-fat, added sugars, and sodium intake during the second, third, and the fourth scenarios.

**Results:**

According to the norm, in the second scenario, the majority of the products presented a label for energy (52.6%) whereas in the third (56.4%) and fourth (61.2%) scenarios were for sodium. In contrast, trans-fat was the least labeled nutrient in all the scenarios (from 2.1 to 4.1%). In the fourth scenario, we observed a reduction of the intake of energy intake to 15.4% as well as saturated fat (− 20%, CI 95% − 18.4; − 21.6), trans-fat (− 8.2%, CI 95% − 6.4; − 10.1) and sodium (− 12.7%, CI 95% − 11.3; − 14.1). The most important reduction was observed for added sugars intake (until − 54.1%, CI 95% − 51; − 57.1).

**Conclusions:**

FOPWL could be an effective strategy to decrease energy consumption and nutrients of concern. If consumers use the FOPWL, it would be an important change in critical nutrients intake. These results support that FOPWL might help the Mexican population to choose healthier nutrition alternatives.

**Supplementary Information:**

The online version contains supplementary material available at 10.1186/s41043-023-00462-7.

## Background

Non-communicable diseases (NCDs) are the leading cause of death worldwide [1] and contribute to more than half of the global burden of disease [2]. Unhealthy diets, typically high in sugars, sodium saturated and trans-fats, low in fruits and vegetables and high-sugar drinks, are one of the main risk factors for ill health [2]. In 2017, 11 million deaths worldwide of which 22% among adults, mostly those caused by NCDs like type 2 diabetes, cardiovascular disease and cancers were attributable to unhealthy diets [3]. In Mexico, according to the National Health and Nutrition Survey (ENSANUT by its acronym in Spanish, 2018) [4], there was an increase in the prevalence of overweight and obesity (from 71.3 to 75.2%) between 2012 and 2018 and the proportion of adults with type 2 diabetes also increased from 9.2 (6.4 million) to 10.3% (8.6 million) in the same period. Besides, diabetes is the main cause of disability and the third cause of mortality in Mexico [4].

The high intake of sodium, added sugars, saturated and trans-fats has been associated with increased risk for NCDs [5]. High levels of this dietary components are found in processed and ultra-processed food and beverages, as defined by the NOVA food classification [6]. This food classification is a system that categorizes foods according to the nature, extent and purpose of food processing, rather than by nutrients [6]. In Mexico, added sugars and saturated fats contribute 12.5 and 11.2% (respectively) to total energy intake [7] Therefore, it is important to reduce the intake of these nutrients and to look for strategies to help the population decrease their intake.

However, in the last decades, diet went through major changes, and the energy intake per capita increased by 580 kcal/day globally [8, 9]. The changes are attributed to the increase in processed and energy-dense food [8]. Mexico is not an exception and has followed the same trend of ultra-processed food; according to NOVA classification, they are the food group that “use many ingredients including food additives that improve palatability, processed raw materials and ingredients that are rarely used in home cooking” [6]. In 2013, Mexico had the highest sales of ultra-processed foods among Latin American countries and the fourth worldwide [10]. Furthermore, from 1984 to 2016 [11], the energy contribution of ultra-processed foods and processed food to household purchases increased from 10.5 kcal to 23.1% and from 5.7 to 6.5% kcal, respectively. In contrast, unprocessed or minimally processed foods decreased from 69.8 to 61.4% kcal [11].

Guidance to choose healthy foods using the Front-of-Pack Warning Label (FOPWL) system for processed food is an important cost-effective policy to reduce the intake of critical nutrients [12–16] along with educational nutrition programs. The FOPWL is part of a group of strategies established to improve the food environment. In Mexico, the official FOPWL Regulation (Official Mexican Standards, NOM-51, for its Spanish acronym) went into effect October 2020 and determines the eligibility of foods and the thresholds for the content of energy, saturated fat, trans-fat, added sugars and sodium in processed food per 100 g or ml, based on the Pan American Health Organization (PAHO) nutrient profile model. To give time to the food industry to adapt its products according to the standard, the implementation will be carried out in 3 phases. The first phase of the regulation began in October 2020, the second will begin in October 2023 and the last phase will begin in October 2025 although the thresholds for them will become progressively stricter over 5 years across the phases.

Under the NOM-51, processed foods must include warning labels (WL) when the content of the previous nutrients of interest exceeds the thresholds. For example, a product will bear the “Excess sugar” and “Excess saturated fat” label if the product’s sugar or saturated fat content is 10% or more of the total calories. The “Excess trans-fat” label will be used if trans-fat content is 1% or more. For the “Excess sodium,” label, criteria include 1 mg or more of sodium per calorie or 300 mg or more of sodium in beverages and packaged foods; for non-caloric beverages, 45 mg or more of sodium [17]. In consequence, a product within a food group with more WL is less healthy for consumption. Thus, it is expected that consumers with different levels of literacy will understand easily the nutritional content and will discriminate between a healthy and unhealthy product allowing them to make more informed choices in a practical way [18]. In Latin American countries, a number of studies evaluated the FOPWL impact on awareness [19, 20], understanding [20–22], acceptability [23], use [19] and the effects on healthfulness perceptions [24, 25]. However, only one Chilean study evaluated the impact of FOPWL on household beverage purchases [16]. Besides, FOPWL is a public policy whose compliance must be monitored and evaluated either by the government or by academia. This is an effort to hypothetically evaluate the impact of the policy on the consumption of energy and critical nutrients that could be observed if FOPWL were used by the Mexican population. Previous studies have proved that modeling is useful to accurately estimate the nutrient intakes when processed food is replaced using healthier alternatives [26–28]. These studies are frequently used to anticipate dietary changes before they are implemented in populations like in Mexico. Therefore, we aimed to assess the potential of the FOPWL on Mexican population’s intake of critical nutrients and energy by replacing products with several WL with those with a fewer number of them or with non-processed food in four hypothetical scenarios represented the phases of the regulation.

## Methods

### Study design and sample

The National Health and Nutrition Survey of 2016 (ENSANUT 2016, for its Spanish acronym) is a cross-sectional, multistage, probabilistic, stratified, cluster survey with national and urban/rural representativeness [29], carried out between May and September of 2016. Data on 29,795 individuals from 9474 randomly selected households, were collected with a response rate of 78%. The main objective was to characterize the health and nutritional status of the Mexican population [29]. The specific survey methodology and the sampling procedures were described previously [29]. All participants signed informed consent, and the study protocol was approved by the Ethics, Research and Biosafety Committee of the National Institute of Public Health (INSP-Spanish acronym).

Dietary information was obtained for a random subsample of ∼ 15% (*n* = 4341). For the present analysis, we included adults, adolescents, school-aged children, and preschool children. We excluded pregnant and lactating females (*n* = 89), participants with an extreme energy intake (± 3 standard deviations of the ratio of energy intake and energy requirement, *n* = 64) and breastfed children (*n* = 4). Thus, the analytical sample included 4184 participants.

### Assessment of dietary intake

Dietary data were collected using a standardized 24-h dietary recall applied by trained personnel. An automated software was used to collect this data, which was an adapted version of the original method created by the United States Department of Agriculture [30]. To capture more accurately the food intake and avoid underestimation, a multiple five-pass methods was used [30]. Participants reported all food and beverages consumed in the previous 24 h period, and the type and amount of food items consumed were recorded. Participants under 15 years were assisted by the person responsible for food preparation in their household.

To estimate nutrient content, first food and beverages reported in the 24-h recalls were classified in processed and unprocessed food according to NOVA classification [6] (See Additional file 1: Table 1). For unprocessed food, we estimated energy and nutrient content using the 2016 INSP food composition table (Nutrient Database, Compilation of the Mexican National Institute of Public Health, unpublished material, 2016). Most processed food in the 24-h recall were not at the brand level, with some exceptions such as boxed cereals and some cereal-based desserts. For non-branded products, such as chips, we selected the best-selling brand in Mexico using the Nielsen Consumer Panel (2016), which is a global leader in retail measurement services. For the selected product at the brand level, the nutritional content was matched using the 2017 INFORMAS database [31] (a database of food composition of package foods and beverages that were available in the main chains of retail stores in Mexico). We were able to match 82% of products to their specific nutrition composition. If the nutritional content of the selected brand was not available, an average of the brands in the database was estimated. For the remaining products, for which no brand was available, we used the nutritional content of the 2016 INSP food composition table.

### The Mexican regulation

Details of the official FOPWL Regulation (Official Mexican Standards, NOM-51) were described previously [17]. The FOPWL consists of a black octagon with white borders which highlights that the product has an excess in the critical nutrients (added sugars, sodium, trans and saturated fat) and energy per 100 g using the wording “Excess in” in front of the package (Table 1). To give time to the food industry to adapt its products according to the standard, the implementation will be carried out progressively in 3 phases. The first phase of the regulation began in October 2020, the second will begin in October 2023 and the last phase will begin in October 2025.

**Table 1:** Criteria of the front-of-pack warning label (FOPWL) regulation, NOM-051. Mexico 2019

Thresholds in 100-g solid products and 100 ml liquid products						
Phase *	Date	Energy	Added sugars	Saturated fats	Trans-fats	Sodium
First	Begin October 2020	≥ 275 kcal total (solid product) ≥ 70 kcal total or ≥ 10 kcal from free sugars (liquid product)	≥ 10% total kcal from free sugars Beverages with < 10 kcal free sugars are exempted from warning label	≥ 10% total kcal from saturated fats	≥ 1% total kcal from trans-fat	≥ 350 mg (solid products) Calorie- free beverages: ≥ 45 mg
Second	Begin October 2023	≥ 275 kcal total (solid product) ≥ 70 kcal total or ≥ 8 kcal from free sugars (liquid product)	≥ 10% total kcal from free sugars	The same threshold values	The same threshold values	≥ 1 mg sodium per kcal or ≥ 300 mg (solid products) Calorie-free beverages ≥ 45 mg
Third	Begin October 2025					
Warning labels (WL)	A product will bear a WL if exceeds the thresholds stablished in each phase	EXCESO CALORÍAS (Excess Calories)	EXCESO AZÚCARES (Excess Sugars)	EXCESO GRASAS SATURADAS (Excess Saturated Fats)	EXCESO GRASAS TRANS (Excess Trans-Fats)	EXCESO SODIO (Excess Sodium)
Warning symbols for energy and critical nutrients						

^*^The evaluation of critical nutrients to determine if a product would bear a label varied across the phases of the norm. During the first and second phase of the norm, the evaluation was as follows: if added sugars was added, sugars and calories were evaluated. If fats were added, saturated fats, trans-fats and calories were evaluated; finally, if sodium was added, only sodium was evaluated. In the second and third phases, the thresholds were the same. For the third phase, all critical nutrients must be evaluated if the product contained at least one of them

Packaged food with added salt, fat, or sugars was required to carry the FOPWL if the content of them exceeded the established thresholds in the norm, the details of each phase are presented in Table 1. The evaluation of critical nutrients to determine if a product would bear a label varied between the phases of the norm, the thresholds become stricter across the phases. Under the regulation, processed foods must include warning labels (WL) when the content of the nutrients of interest exceeds the thresholds described in Table 1. During the first and second phase of the norm, the evaluation was as follows: if added sugars was added, sugars and calories were evaluated. If fats were added, saturated fats, trans-fats and calories were evaluated; finally, if sodium was added, only sodium was evaluated. In the second and third phases, the thresholds were the same. For the third phase, all critical nutrients must be evaluated if the product contained at least one of them. Three types of products were exempted from labeling: unpackaged products, culinary ingredients (e.g., oils and salt), and products for infants and young children, including infant formula.

### FOPWL assessment and replacement

We estimated the amount and type of WL each processed food would have, according to the FOPWL Regulation. Accordingly, these items could have zero to five WL. For the replacement, we consider two criteria: the number of labels and the type of processed food. Thus, we created 18 food groups, within which the number of WL varied (Table 2). Given this variation, we used products with 0 or 1 WL to replace products with 2 or more labels. However, there were food groups in which there were no products with 0 or 1 WL; therefore, we selected the product with less WL. For some products, we were unable to find a suitable alternative, for which, we simulated the effect of reducing product’s intake by 5%. This 5% approach is a hypothetical scenario. For example, in the food group “Salted bread” for the product rye bread which has 0 labels, we did not find a processed product to replace it with.

**Table 2:** Number of warning labels (WL) by food groups for processed food (*n* = 420)

Food groups	Number of WL by phases of the NOM-051		
	First	Second	Third
	Min–Max	Min–Max	Min–Max
Tubers, beans, legumes, cereals, corn and fours	0–3	0–3	0–3
Tortillas and corn products	0–3	0–3	0–3
Salted bread	0–3	0–3	0–3
Breakfast cereal products, cereal bars, granola	1–4	1–4	1–4
Milk and dairy products	0–4	0–4	0–4
Cheese (products)	0–4	0–4	2–4
Processed Meat (fresh and cured sausages)	1–3	1–3	1–4
Fish and shellfish (fresh or frozen)	0–2	1–2	1–2
Soups, creams and broths	1–2	1–3	1–3
Industrial food	1–3	1–3	1–3
Dressings, condiments, stock cubes and powders	0–3	1–3	1–3
Sauces (water based and emulsions)	1–3	1–3	1–4
Snacks (savory and sweet)	0–4	0–4	0–4
Sweet bread, pastry, desserts and cookies	1–4	1–4	1–4
Processed fruits and vegetables	0–2	1–2	0–2
Non-alcoholic beverages	0–3	0–3	0–4
Oils, fats and fat containing spreads	2–3	2–3	2–3
Sweeteners, condensed milk, jelly, milk jam	0–3	0–3	1–3

Four scenarios were established to evaluate the replacement and they are given as follows: Scenario 1 represents the current consumption of the Mexican Population, scenario 2 simulates the replacement during the first phase of the norm and each product was replaced by a similar one with less WL and the same amount in grams, scenario 3 simulates the replacement during the second phase of the norm, and scenario 4 simulates the replacement during the third and last phase of the norm. In this last scenario, we also aimed to replace packaged items with WL with non-processed food and beverages. For example, mayonnaise was replaced in the second and third scenarios with mayonnaise light. However, in the fourth scenario, there was no option to replace it with; thus, we reduced its consumption by 5%.

### Sociodemographic variables

We included the following sociodemographic characteristics: sex, age group, socioeconomic status (SES), locality (rural: < 2 500 inhabitants/urban: ≥ 2 500 inhabitants) and geographic region (North, Center, South and Mexico City). We created 4 age groups: adults (≥ 20 years), adolescents (12–19 years), school-aged children (5–11 years) and preschool children (1–4 years). The SES variable was created using principal component analysis which included household characteristics and goods. This variable was divided into tertiles and used as a proxy for low, medium, and high SES.

### Statistical analysis

Energy and nutrient intake of the population was estimated and presented in means and 95% confidence intervals (95% CI). Furthermore, intake was stratified by sociodemographic characteristics. Finally, we estimated the mean percentage change of energy, saturated fat, trans-fat, added sugars and sodium intake for the second, third and fourth scenarios. For that, first we estimated the intake differences of critical nutrients, we used the first scenario as reference and compared with the second, third and fourth scenarios, and then these differences were transformed into percentages. All statistical analyses were performed using Stata version 14 (College Station, TX, USA), and the SVY module was used to adjust the complex sampling design of ENSANUT 2016.

## Results

Baseline sociodemographic characteristics of participants are described in Table 3. Most of the participants were adults (60.3%), women (51.4%), from the urban area (74.9%), south region (36.1%) and the high socioeconomic level (48.3%).

**Table 3:** Sociodemographic population characteristics. National Survey of Health and Nutrition (ENSANUT) 2016, Mexico (*n* = 4184)

Sociodemographic Characteristics	*n*	%*
*Sex*		
Men	1882	48.6
Women	2302	51.4
*Age group*		
Preschool-aged children (1–4 y)	523	8.2
Scholar-aged children (5–11 y)	1085	14.6
Adolescents (12–19 y)	1230	16.9
Adults (≥ 20 y)	1346	60.3
*Region*		
North	925	22.1
Center	1325	31.7
Mexico State and Mexico’s City	425	10.1
South	1509	36.1
*Locality*		
Rural	2216	25.1
Urban	1968	74.9
*Socioeconomic level*		
Low	1455	21.6
Medium	1500	30.1
High	1229	48.3

*Data adjusted by survey design

**Fig. 1:**
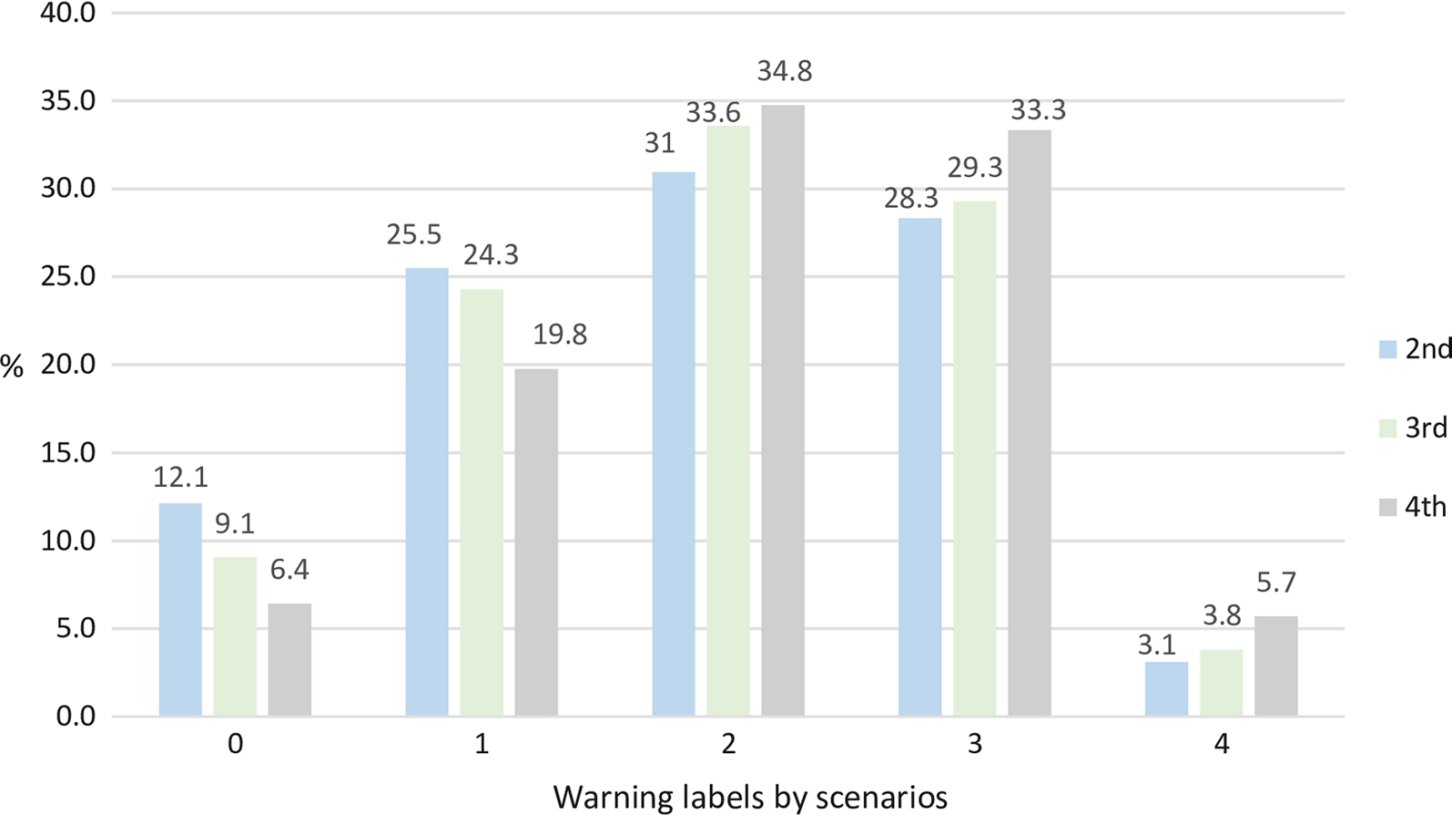
Percentage of warning labels in processed food by scenarios according to the criteria of the front-of-pack warning label (FOPWL) regulation, NOM-051. Mexico 2019

Concerning food and beverages FOPWL evaluation, 1527 unique products were reported in the 24-h recalls, 27.5% (*n* = 420) of which were processed products and candidates to be evaluated by the NOM-051. The number of WL by norm scenarios represented in percentage are shown in Fig. 1. According to the criteria of the norm, in the second scenario, 12.1% of the food products would not bear a WL, this percentage decreased gradually across phases until 6.4% in the fourth scenario. The products that would have only one label decreased between scenarios (from 25.5 to 19.8%); thus, the products that would declare 2, 3 and 4 labels increased between the second and the fourth scenarios from 31 to 34.8%, 28.3 to 33.3% and 3.1 to 5.7%, respectively. In this study, none of the products would declare WL for all the critical nutrients.

**Fig. 2:**
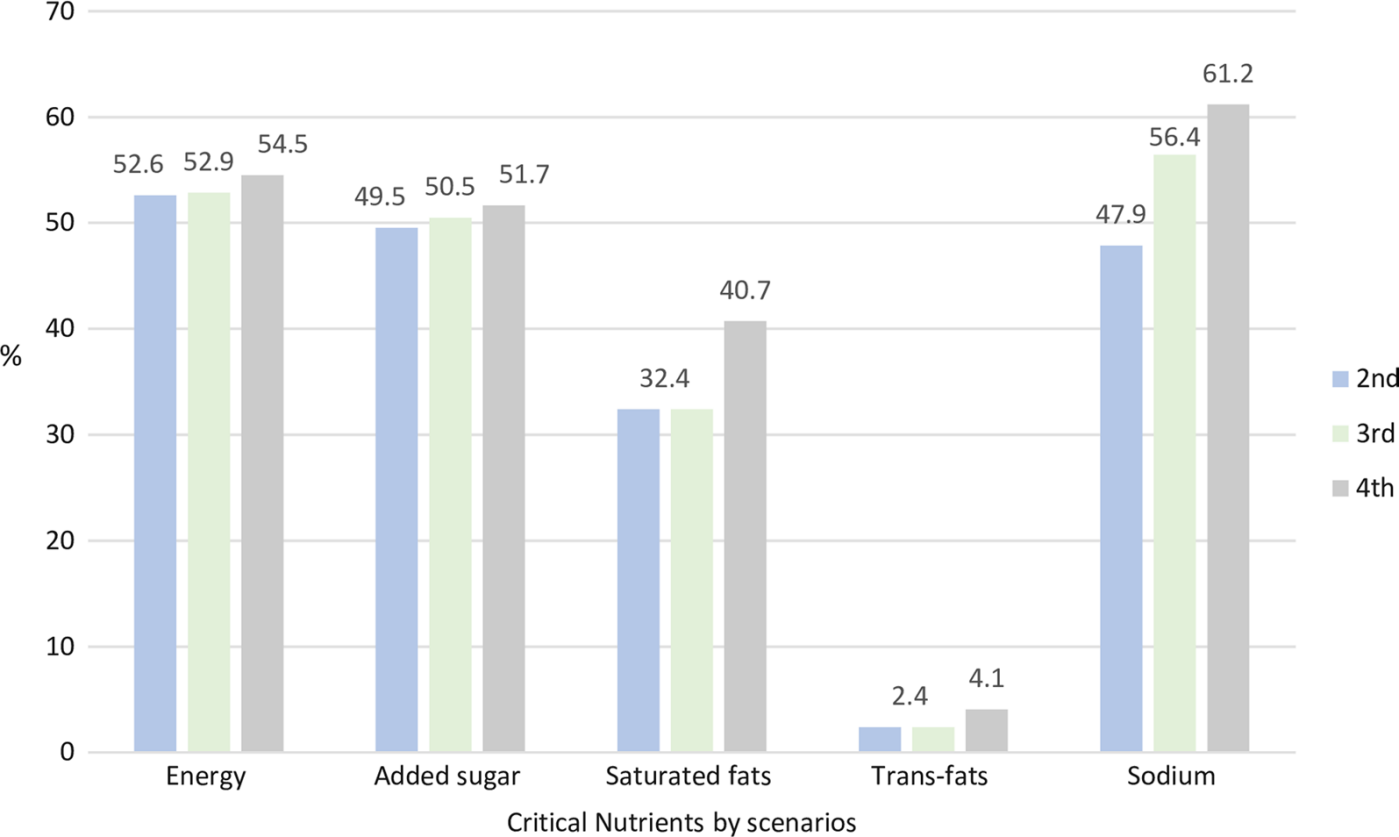
Percentage of warning labels by critical nutrient in processed food according to the criteria of the front-of-pack warning label (FOPWL) regulation, NOM-051. Mexico 2019

During the second scenario, the most labeled nutrient was energy (52.6%) and in the third (56.4%) and fourth scenarios was sodium (61.2%), while trans-fat was the least, from 2.4 to 4.1% in the second and fourth phases, respectively. The WL for saturated fat (32.4%) and added sugars (2.4%) did not change between the second and third scenarios of the norm only in scenario 4 (Fig. 2). Regarding the replacement of food and beverages according to the FOPWL regulation, among the products consumed by the Mexican population, the replacement of the products increased across the scenarios in 55.9%, 57.6% and 82% in the second (first phase of the norm), third (second phase of the norm), and fourth scenarios (third phase of the norm), respectively.

Mean intakes of energy, saturated fat, trans-fat, added sugars and sodium before and after the replacement of food according to the FOPWL criteria for all the population are presented in Table 4. We observed changes in the critical nutrients consumption after the replacement. In general, for all the Mexican population, we found an important decrease in the critical nutrients intake when we replaced food according to the FOPWL criteria during the second, third and fourth scenarios. When we stratified the critical nutrient intake by socioeconomic characteristics, we observed the same behavior (See Additional file 2: Table 2).

**Table 4:** Critical nutrient intake before and after the replacement with FOPWL criteria in the Mexican population. Mexico, ENSANUT 2016

Critical nutrients	Mexican population		
	Mean	95% CI	
*Energy (kcal)*			
Scenario 1	1817	1751	1882
Scenario 2	1610	1551	1668
Scenario 3	1606	1548	1665
Scenario 4	1534	1476	1592
*Saturated fat (g)*			
Scenario 1	20.4	19.4	21.5
Scenario 2	16.3	15.5	17.2
Scenario 3	16.2	15.4	17.0
Scenario 4	15.7	14.9	16.5
*Trans-fat (g)*			
Scenario 1	0.2	0.1	0.2
Scenario 2	0.1	0.1	0.2
Scenario 3	0.1	0.1	0.2
Scenario 4	0.1	0.1	0.1
*Sodium (mg)*			
Scenario 1	8809.0	8012.4	9605.7
Scenario 2	7695.9	7020.6	8371.1
Scenario 3	7677.7	7002.1	8353.3
Scenario 4	7444.7	6768.0	8121.4
*Added sugar (g)*			
Scenario 1	57.2	53.1	61.3
Scenario 2	27.9	24.5	31.2
Scenario 3	27.9	24.5	31.2
Scenario 4	23.8	20.9	26.8

FOPWL, Front-of-Pack Warning Label; Scenario 1, Current intake of Mexican population; Scenario 2, Energy and nutrient intake after the simulation during the first phase of the norm; Scenario 3, Energy and nutrient intake after the simulation during the second phase of the norm; Scenario 4, Energy and nutrient intake after the simulation during the third phase of the norm; Data adjusted by survey design

**Fig. 3:**
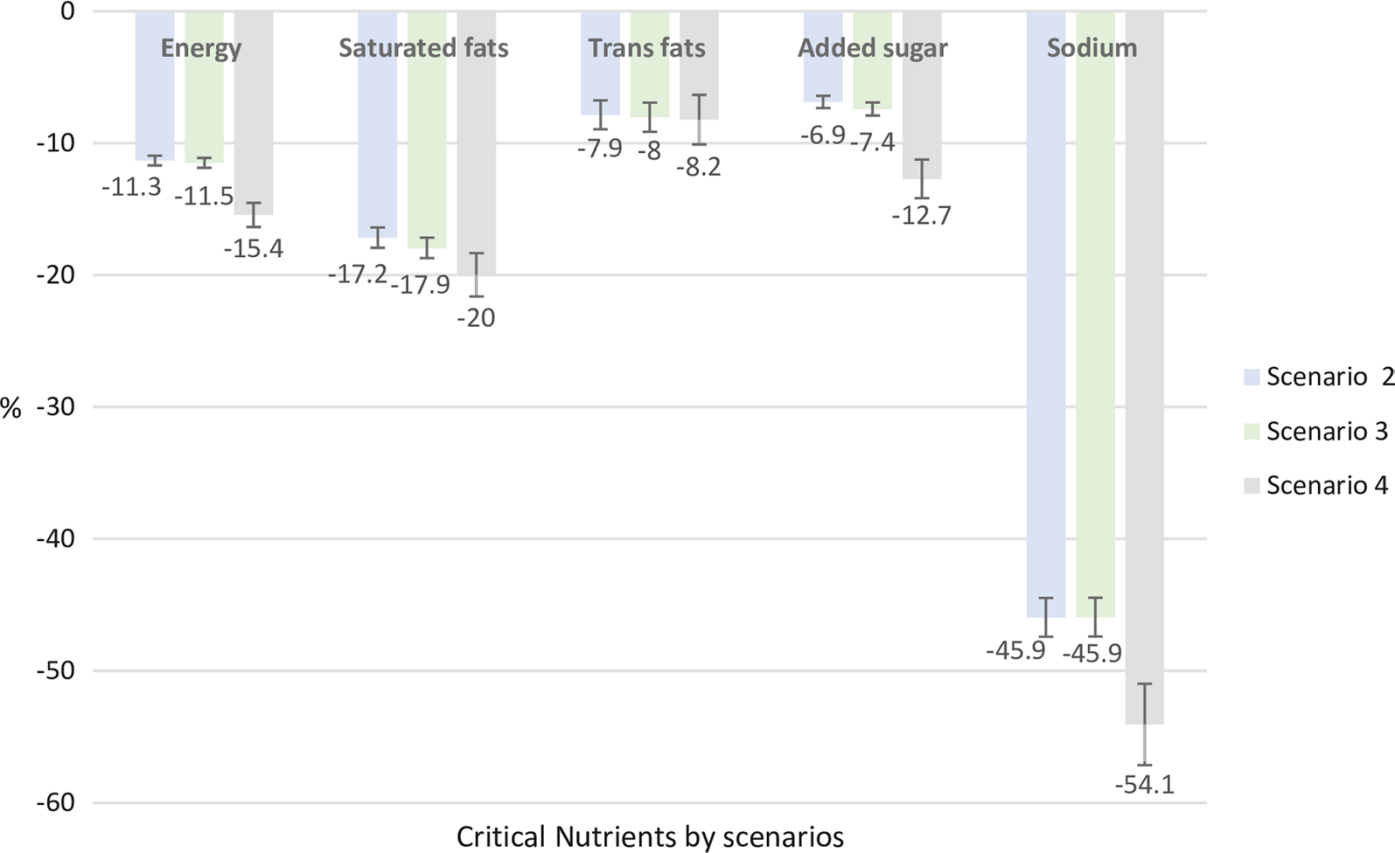
Percentage change in the mean intake of energy, saturated fat, trans-fat, sodium and added sugars after the replacement with FOPWL criteria in all the Mexican population. Mexico, ENSANUT 2016. *Comparison: Scenario 1 versus Scenario 2; Scenario 1 versus Scenario 3 and Scenario 1 versus Scenario 4

The mean percentage change of energy, saturated fat, trans-fat, added sugars and sodium intake during the second, third and fourth scenarios was estimated in Fig. 3. During the second and third scenarios, we observed a small or a constant reduction in the consumption for all the critical nutrients across the scenarios. In contrast, in the fourth scenario, we observed the most important reduction for energy (− 15.4%, CI95% − 14.5; − 16.3), saturated fat (− 20%, CI95% − 18.4; − 21.6), sodium (− 12.7%, CI95% − 11.3; − 14.1) and trans-fat intake (− 8.2%, CI 95% − 6.4; − 10.1). Among the nutrients in this scenario, the most important reduction was for added sugars intake (− 54.1%, CI 95% − 51; − 57.1).

## Discussion

In this study, we evaluate the hypothetical impact of the FOPWL regulation, and we observed a reduction of energy, saturated fat, trans-fat, added sugars and sodium intake, after a simulated replacement of commonly consumed foods in the Mexican diet, with products that would have less WL according to the FOPWL criteria in all the population and by socioeconomics characteristics across the three phases of the norm. The most important reduction was observed for added sugars intake. To our knowledge, this is the first study in Mexico to evaluate through food and beverage replacement the potential impact of the FOPWL norm under the assumption that consumers would replace consumed products with those with a fewer WL or with non-processed food.

The results in these analyses are consistent with similar studies. In a previous study also in Mexico [32], processed food was replaced with food that complied with the Mexican Committee of Nutrition Experts (based on the dietary intake recommendations from the World Health Organization) and Federal Commission for the Protection against Sanitary Risks Nutrition Criteria (based on the cutoff points established by the food and beverage industry) and then compared with the baseline consumption [32]. Similar to our study, a reduction was seen for energy (− 5.4%), saturated fat (− 18.9%), trans-fat (− 20%), total sugar (− 36.8%) and sodium (− 10.7%) intake [32]. The criteria for evaluating critical nutrients were different from the FOPWL used for this analysis, hence the differences in reductions. Besides, an increase in fiber intake was shown; however, we did not evaluate fiber and micronutrients intake since our focus was in the assessment on the critical nutrients that FOWPL regulation involved. However, it would be likely that in our replacement simulation, fiber would also increase since we increased the consumption of foods rich in dietary fiber during the replacement.

In this study, the greatest reduction was observed for added sugars in all the population and by socioeconomic characteristics. A possible explanation is the high intake of sugar-sweetened beverages in the Mexican population. In 2012, added sugars consumption represented 12.5% of total energy and 70% of them came from sugar-sweetened beverages [33]. Due to the high intake, Mexico is the first in the ranking of mortality and morbidity attributable to these beverages [34, 35]. Further, in a recent national survey ENSANUT 2018, adults (85.8%), adolescents (85.7%), school-aged children (85.7%), preschool-aged children (83.3%) reported an important consumption of these beverages [36], despite the efforts to discourage their consumption in the last years.

The impact of Chile’s Law of Food Labeling and Advertising which includes the FOPWL was evaluated on household beverage purchases [16]. Using fixed-effects models they compared the observed volume and calorie sugar content in beverage purchases after the regulation, with a counterfactual based on pre-regulation trends. The volume of high-in beverage purchases decreased by 22.8 ml/capita/day (95% CI − 22.9 to − 22.7; *p* < 0.001) or 27% after the regulation was implemented. Nonetheless, it is not certain whether the observed changes were due to reformulation or a real change in consumer behavior.

In the Netherlands, in young adults (aged 19–30 years), a substitution of current food intake with those that complied with the Choices Program (an international nutrient profiling system to determine whether foods are eligible to carry a healthier option label) was conducted [37]. After the replacement, they observed that median energy intake was reduced by 16% as well as for nutrients with a maximal intake limit (from 23 for sodium to 62% for trans-fat). Contrary to our study, they showed a greater reduction in trans-fat intake in relation to other nutrients. This may be explained because of the differences in dietary patterns or higher content of this nutrient in Dutch-processed food. Besides, in this study, the trans-fat intake could underestimate since the labeling regulation in force in 2016 presented some deficiencies that allowed to declare 0 g of trans-fats when they were below 0.5 g per portion.

Furthermore, during the third scenario, the cutoff points for trans-fat and saturated fat did not change; if the cutoff points for them would be stricter in the last phase of the norm, there would be more products to replace hence we could observe a greater reduction in these nutrients. Moreover, the trans-fats intake might be already conditioned by other efforts such as the Action Plan to eliminate industrially produced trans fatty acids 2020–2025 and the Replace initiative from the World Health Organization (WHO), where the elimination of trans-fats in industrial food processes is proposed [38]. However, we expect that the reductions shown for all the nutrients in this study will be more important since the food industry will have the opportunity to modify the nutritional content of their products according to the cutoff points of the norm. Thus, even if consumers did not change their dietary choices, in expectation they would consume less amount of energy, saturated fat, trans-fat, added sugars and sodium from the newly reformulated products.

The Mexican Government has implemented different strategies to enhance the dietary quality of the population, such as taxes on junk food and sugary beverages, banning the sale of processed foods and sugary beverages in schools, establishing restrictions for advertising directed to children, implementing physical activity programs and bike lanes in the community, and making public water fountains more available [39–43]. Unlike previous measures, the FOPWL regulation has the advantage that can be monitored and enforced and have the possibility to change the cultural norms that regulate consumer demand, thus food supply over time [18].

Our assumption regarding product substitution is in line with existing empirical results in Chile. Specifically, evidence from Chile shows that WL mainly encourage product substitution within the same food category [44]. Moreover, some studies provide evidence of the underlying mechanisms behind the purchase adjustments after the WL implementation in Chile, accounting for the simultaneous response to this policy by consumers and producers. For example, Barahona et al., in the case of breakfast cereals in Chile, showed product substitution between labeled and unlabeled cereals due to the interplay between updates in healthfulness beliefs by consumers because of the information conveyed by WL and price adjustments by producers in response to the cost linked to product reformulation [45]. Meanwhile, Pachali et al. found, likewise for breakfast cereals in Chile, that reductions in the nutrients of concern resulted from consumers substituting labeled for unlabeled cereals [46]. Moreover, Pachali et al. showed this substitution was reinforced by a price decrease across unlabeled cereals [46]. The authors pointed out that this price decrease was a producers’ response to the new composition of consumers demanding unlabeled cereals that were more price-sensitive than previous consumers before the WL implementation [46]. However, it is worth noting that the expected WL effect is likely to be food group specific in light of the findings by Araya et al., who showed no clear change in purchases of chocolates and cookies in Chile [47]. Evidence in Mexico in the context of the sugar-sweetened-beverage tax showed that sugar content is a demand driver, aside from prices and products’ brand, so consumers are likely to decrease their demand when producers reformulate their products in case this reformulation leads to a sweetness reduction (i.e., sugar reduction with no substitution for non-nutritive sweeteners) [48]. Thus, if WL encouraged reformulation in Mexico, consumers would likely adjust their demand downwards, at least for sugar-sweetened beverages. Finally, aside from reformulation, producers might respond to WL by removing/launching products. However, evidence from South Africa shows that this update in producers’ product portfolios has a minor contribution to the overall purchase changes linked to sugar-density taxes on sugar-sweetened beverages, which encourage reformulation as WL does [49]. In light of all these results, we consider our product substitution assumption consistent with existing evidence. However, we acknowledge our estimate could be conservative by overlooking producers' response to WL (i.e., reformulation and price adjustments) and how consumers react to this response. When the FOPWL regulation would be finished, there will be more food products options, thus more variability to select food products for the replacement; unfortunately, this was not possible due to the scope of this work and the phase of the FOPWL regulation.

Previous studies have proved that modeling is useful to accurately estimate the nutrient intakes when processed food is replaced using healthier alternatives [26–28]. These studies are frequently used to anticipate dietary changes before they are implemented in populations like in Mexico. Thus, the data could be turned into nutrition policies and help decision-makers to design policies based in evidence that would help to decrease diet and non-communicable diseases.

Healthy foods are often unaffordable or unavailable, the increase in urbanization causes population to change their dietary habits, to move away from producing and cooking their own food to prepared foods (high in sugar, salt and fat) [50]. Besides, marketing influences food choices, and unhealthy foods are an important target, especially among children [51]. Therefore, legislation and policies should focus on regulating prices, production, processing and distribution to make healthy foods more accessible as well as to restrict marketing to reduce the promotion of unhealthy food. FOPWL regulation is an important strategy to help population to identify unhealthy food in a simple way [52], along with public health interventions that can emphasize the importance of balanced diets on maintaining good health. The community health programs can educate children, parents, teachers and community leaders about the impact of their nutrition choices on health [53, 54].

Furthermore, through this simulation, we can estimate the potential impact of the FOPWL explained uniquely by change in consumer’s behavior. In Mexico, when the implementation of the regulation is completed, it will be difficult to determine if intake changes are because of consumer’s behavior, product reformulation, changes in marketing, other policies or the interaction of all of them.

This study presented some limitations. First, it is uncertain if the Mexican population will replace products with less WL or with non-processed products. This is possible since the population choices and intakes are determined by an array of factors, and not only on the presence or absence of WL [55]. Moreover, ENSANUT 2016 is a cross-sectional observational dataset; for this analysis, we used self-reported intake data which may be affected by measurement error. Further research is necessary to know if this error is randomly distributed through the different subgroups or whether it affects systematically certain subpopulations. However, in the Mexican population, this database is the most comprehensive nationally representative data to study dietary intake. Another limitation may be the availability of products presented in 2017 from the INFORMAS dataset [31]. Further, despite the results observed, we were confronted an important limitation to replace sugar-sweetened beverages, which was the low variability of the number of warning labels in this group of drinks between 0 and 2 WL. During the second and third scenarios, the replacement was using the same drink; thus, the reduction would be greater in the third scenario if more options were available. Finally, our results will need to be corroborated by future observational studies when the implementation of the regulation will be finished.

## Conclusion

In this study, we showed that the FOPWL could be an effective strategy to decrease the consumption of added sugars, trans-fat, saturated fat added sugars and sodium intake if replacement of products is done by the population. The use of FOPWL would lead to an important change in critical nutrients intake previously associated with cardio metabolic diseases, and this is particularly important in Mexico considering the high rates of overweight and obesity and the complication of these diseases. It is important to promote the use of warning labels to discourage the use of processed food intake. However, this must go with nutritional education to raise awareness of the implications of a good diet in the population. Besides, food environment, food system, and nutritional education strategies should encourage the intake of non-processed food or minimally processed food.

### Supplementary Information

**Additional file 1: Table 1.** Categories of processed food according to NOVA classification.

**Additional file 2: Table 2.** Critical nutrient intake before and after the replacement with FOPWL criteria by sociodemographic characteristics. Mexico, ENSANUT 2016.

## Data Availability

The datasets generated and/or analyzed during the current study are not publicly available due to the request of the funder. The funder has requested to safeguard the information for five years.

## Abbreviations

FOPWL: Front-of-pack warning labels
NOM-051: Mexican Official Standard 051
CI 95%: Confidence intervals
NCDs: Non-communicable diseases
PAHO: Pan American Health Organization
WL: Warning labels
ENSANUT 2016: The National Health and Nutrition Survey of 2016, Spanish acronym
INSP: The National Institute of Public Health, Spanish acronym
