# Supplementary material for: Hypothetical impact of the Mexican front-of-pack labeling on intake of critical nutrients and energy
Source: J Health Popul Nutr. 2023 Nov 8;42:124. doi: 10.1186/s41043-023-00462-7 (PMC10631037; doi:10.1186/s41043-023-00462-7)
Supplement: Supplementary file 1 — Additional file 1: Table 1. Categories of processed food according to NOVA classification. [file 41043_2023_462_MOESM1_ESM.docx]

**Additional file 1: Supplementary Table 1. Categories of processed food according to NOVA classification.**

| **Group** | **Definition** | **Purpose of the processing** | **Examples** |
| --- | --- | --- | --- |
| **1. Unprocessed or minimally processed foods** | Unprocessed or natural foods are edible parts of plants or of animals, and also fungi, algae and water, after separation from nature. Minimally processed foods are natural foods altered by processes that include removal of inedible or unwanted parts, and drying, crushing, grinding, fractioning, filtering, roasting, boiling, non-alcoholic fermentation, pasteurization, refrigeration, chilling, freezing, placing in containers and vacuum-packaging. | Ultra-processed foods, are not modified foods but formulations made mostly or entirely from substances derived from foods and additives, with little if any intact Group 1 food. Ingredients of these formulations usually include those also used in processed foods. But ultra-processed products also include other sources of energy and nutrients not normally used in culinary preparations, such as casein, lactose, whey and gluten. Many are derived from further processing of food constituents, such as hydrogenated or interesterified oils, hydrolysed proteins, soya protein isolate, maltodextrin, invert sugar and high-fructose corn syrup. Additives in ultra-processed foods include some also used in processed foods, such as preservatives, antioxidants and stabilizers. Classes of additives found only in ultra-processed products include those used to imitate or enhance the sensory qualities of foods or to disguise unpalatable aspects of the final product. | Seeds, fruits, leaves, stems, roots, muscle, offal, eggs, milk vegetables, nuts, fish, pulses, egs and grains |
| **2. Processed culinary ingredients** | Processed culinary ingredients, are substances derived from Group 1 foods or from nature by processes that include pressing, refining, grinding, milling and drying. | The purpose of such processes is to make durable products that are suitable for use in home and restaurant kitchens to prepare, season and cook Group 1 foods and to make with them varied and enjoyable hand-made dishes and meals, such as stews, soups and broths, salads, breads, preserves, drinks and desserts. They are not meant to be consumed by themselves, and are normally used in combination with Group 1 foods to make freshly prepared drinks, dishes and meals. | Oils, butter, salt, sugar and vinegar |
| **3. Processed foods** | Processed foods, such as bottled vegetables are made essentially by adding salt, oil, sugar or other substances from Group 2 to Group 1 foods. Processes include various preservation or cooking methods, and, in the case of breads and cheese, non-alcoholic fermentation. Most processed foods have two or three ingredients, and are recognizable as modified versions of Group 1 foods. They are edible by themselves or, more usually, in combination with other foods. | The purpose of processing here is to increase the durability of Group 1 foods, or to modify or enhance their sensory qualities. | Bottled vegetables, canned fish, fruits in syrup, cheeses, freshly made breads, tinned fruits, salted nuts and cheese |
| **4. Ultra-processed foods** | Ultra-processed foods, are not modified foods but formulations made mostly or entirely from substances derived from foods and additives, with little if any intact Group 1 food. Ingredients of these formulations usually include those also used in processed foods. But ultra-processed products also include other sources of energy and nutrients not normally used in culinary preparations. Some of these are directly extracted from foods, such as casein, lactose, whey and gluten. Many are derived from further processing of food constituents, such as hydrogenated or interesterified oils, hydrolysed proteins, soya protein isolate, maltodextrin, invert sugar and high-fructose corn syrup. A multitude of sequences of processes (hydrogenation and hydrolysation, extrusion and moulding, and pre-processing for frying) is used to combine the usually many ingredients and to create the final product. | The overall purpose of ultra-processing is to create branded, convenient (durable, ready to consume), attractive (hyper-palatable) and highly profitable (low-cost ingredients) food products designed to displace all other food groups. Ultra-processed food products are usually packaged attractively and marketed intensively. | Soft drinks, sweet or savoury packaged snacks, reconstituted meat products and pre-prepared frozen dishes |

The information was obtained from: Monteiro CA, Cannon G, Moubarac JC, Levy RB, Louzada MLC, Jaime PC. The UN Decade of Nutrition, the NOVA food classification and the trouble with ultra-processing. Public Health Nutrition. 2018;21(1):5-17.
