# Supplementary material for: Hypothetical impact of the Mexican front-of-pack labeling on intake of critical nutrients and energy
Source: J Health Popul Nutr. 2023 Nov 8;42:124. doi: 10.1186/s41043-023-00462-7 (PMC10631037; doi:10.1186/s41043-023-00462-7)
Supplement: Supplementary file 2 — Additional file 2: Table 2. Critical nutrient intake before and after the replacement with FOPWL criteria by sociodemographic characteristics. Mexico, ENSANUT 2016. [file 41043_2023_462_MOESM2_ESM.docx]

**Additional file 2: Supplementary Table 2. Critical nutrient intake before and after the replacement with FOPWL criteria by sociodemographic characteristics. Mexico, ENSANUT 2016.**

|  | **Sex** | | | | |  | **Age groups** | | | | | | | | | | | |
| --- | --- | --- | --- | --- | --- | --- | --- | --- | --- | --- | --- | --- | --- | --- | --- | --- | --- | --- |
| **Critical Nutrients** | **Men** | | | **Women** | | | **Preschool children** | | | **School-aged children** | | | **Adolescents** | | | **Adults** | | |
|  | **Mean** | **95% CI** | | **Mean** | **95% CI** | | **Mean** | **95% CI** | | **Mean** | **95% CI** | | **Mean** | **95% CI** | | **Mean** | **95% CI** | |
| **Energy (kcal)** |  |  |  |  |  |  |  |  |  |  |  |  |  |  |  |  |  |  |
| Scenario 1 | 2011.1 | 1911.0 | 2111.3 | 1633.0 | 1551.4 | 1714.6 | 1034.9 | 943.8 | 1126.0 | 1650.4 | 1566.7 | 1734.1 | 2038.9 | 1915.7 | 2162.1 | 1910.5 | 1814.5 | 2006.5 |
| Scenario 2 | 1781.0 | 1691.1 | 1870.9 | 1447.4 | 1374.8 | 1520.1 | 924.0 | 840.3 | 1007.6 | 1439.6 | 1368.7 | 1510.5 | 1786.3 | 1666.6 | 1906.0 | 1702.6 | 1618.1 | 1787.1 |
| Scenario 3 | 1777.5 | 1687.9 | 1867.1 | 1444.4 | 1371.7 | 1517.1 | 922.9 | 839.5 | 1006.3 | 1434.1 | 1363.3 | 1505.0 | 1783.3 | 1663.6 | 1902.9 | 1699.5 | 1615.4 | 1783.6 |
| Scenario 4 | 1698.8 | 1612.8 | 1784.9 | 1377.8 | 1302.3 | 1453.2 | 845.1 | 765.5 | 924.6 | 1322.4 | 1255.5 | 1389.4 | 1673.7 | 1550.7 | 1796.7 | 1647.7 | 1565.1 | 1730.3 |
| **Saturated fat (g)** |  |  |  |  |  |  |  |  |  |  |  |  |  |  |  |  |  |  |
| Scenario 1 | 21.9 | 20.3 | 23.5 | 19.0 | 17.7 | 20.4 | 13.8 | 12.4 | 15.2 | 20.7 | 19.3 | 22.0 | 24.8 | 22.8 | 26.7 | 20.1 | 18.5 | 21.8 |
| Scenario 2 | 17.8 | 16.5 | 19.0 | 15.0 | 13.9 | 16.0 | 11.0 | 9.9 | 12.1 | 15.9 | 14.9 | 16.9 | 19.0 | 17.4 | 20.7 | 16.4 | 15.2 | 17.7 |
| Scenario 3 | 17.6 | 16.4 | 18.9 | 14.8 | 13.8 | 15.8 | 11.0 | 9.9 | 12.1 | 15.7 | 14.7 | 16.7 | 18.9 | 17.3 | 20.5 | 16.3 | 15.1 | 17.5 |
| Scenario 4 | 17.1 | 15.9 | 18.3 | 14.3 | 13.3 | 15.4 | 10.4 | 9.4 | 11.5 | 14.8 | 13.9 | 15.8 | 17.8 | 16.2 | 19.5 | 16.0 | 14.8 | 17.2 |
| **Trans fat (g)** |  |  |  |  |  |  |  |  |  |  |  |  |  |  |  |  |  |  |
| Scenario 1 | 0.22 | 0.17 | 0.28 | 0.13 | 0.11 | 0.16 | 0.1 | 0.1 | 0.1 | 0.2 | 0.2 | 0.2 | 0.2 | 0.2 | 0.3 | 0.2 | 0.1 | 0.2 |
| Scenario 2 | 0.16 | 0.11 | 0.21 | 0.09 | 0.07 | 0.11 | 0.0 | 0.0 | 0.1 | 0.1 | 0.1 | 0.2 | 0.1 | 0.1 | 0.2 | 0.1 | 0.1 | 0.2 |
| Scenario 3 | 0.16 | 0.11 | 0.21 | 0.09 | 0.07 | 0.11 | 0.0 | 0.0 | 0.1 | 0.1 | 0.1 | 0.2 | 0.1 | 0.1 | 0.2 | 0.1 | 0.1 | 0.2 |
| Scenario 4 | 0.15 | 0.10 | 0.20 | 0.09 | 0.07 | 0.11 | 0.0 | 0.0 | 0.1 | 0.1 | 0.1 | 0.2 | 0.1 | 0.1 | 0.2 | 0.1 | 0.1 | 0.2 |
| **Added sugars (g)** |  |  |  |  |  |  |  |  |  |  |  |  |  |  |  |  |  |  |
| Scenario 1 | 65.6 | 59.5 | 71.7 | 49.2 | 44.0 | 54.5 | 32.2 | 27.8 | 36.7 | 49.4 | 44.2 | 54.7 | 63.0 | 58.4 | 67.7 | 61.1 | 54.7 | 67.6 |
| Scenario 2 | 30.2 | 25.6 | 34.8 | 25.6 | 20.7 | 30.6 | 15.9 | 12.9 | 19.0 | 23.9 | 20.3 | 27.6 | 26.9 | 23.6 | 30.2 | 30.8 | 25.5 | 36.2 |
| Scenario 3 | 30.2 | 25.6 | 34.9 | 25.6 | 20.7 | 30.6 | 15.9 | 12.9 | 19.0 | 23.9 | 20.3 | 27.6 | 26.9 | 23.6 | 30.2 | 30.8 | 25.5 | 36.2 |
| Scenario 4 | 25.9 | 22.3 | 29.6 | 21.9 | 17.1 | 26.6 | 12.4 | 9.6 | 15.3 | 18.2 | 14.9 | 21.4 | 22.4 | 19.2 | 25.7 | 27.3 | 22.6 | 32.0 |
| **Sodium (mg)** |  |  |  |  |  |  |  |  |  |  |  |  |  |  |  |  |  |  |
| Scenario 1 | 9264.7 | 8182.7 | 10346.7 | 8377.6 | 7311.7 | 9443.4 | 4461.8 | 3808.0 | 5115.6 | 6788.7 | 5760.8 | 7816.7 | 10070.5 | 8676.0 | 11465.0 | 9587.3 | 8462.7 | 10711.8 |
| Scenario 2 | 8383.3 | 7385.1 | 9381.4 | 7044.9 | 6314.7 | 7775.1 | 4268.4 | 3622.2 | 4914.7 | 5345.6 | 4605.8 | 6085.4 | 8452.8 | 7317.3 | 9588.2 | 8559.8 | 7636.5 | 9483.1 |
| Scenario 3 | 8362.6 | 7363.9 | 9361.3 | 7029.2 | 6299.0 | 7759.3 | 4264.1 | 3618.3 | 4909.9 | 5314.8 | 4576.0 | 6053.6 | 8437.9 | 7301.3 | 9574.4 | 8541.8 | 7618.4 | 9465.2 |
| Scenario 4 | 8109.8 | 7116.8 | 9102.9 | 6814.9 | 6077.2 | 7552.6 | 4082.0 | 3447.7 | 4716.2 | 5071.5 | 4329.8 | 5813.3 | 8167.2 | 7028.2 | 9306.2 | 8314.4 | 7393.1 | 9235.6 |

FOPWL: Front-of-Pack Warning Label. Scenario 1: Current intake of Mexican population; Scenario 2: Energy and nutrient intake after the simulation during the 1st phase of the norm; Scenario 3: Energy and nutrient intake after the simulation during the 2nd phase of the norm; Scenario 4: Energy and nutrient intake after the simulation during the 3rd phase of the norm.

**Additional file 1: Supplementary Table 1. Critical nutrient intake before and after the replacement with FOPWL criteria by sociodemographic characteristics. Mexico, ENSANUT 2016.**

|  |  | **Locality** | | | |  |  | **Socioeconomic status** | | | | | | | |
| --- | --- | --- | --- | --- | --- | --- | --- | --- | --- | --- | --- | --- | --- | --- | --- |
| **Critical Nutrients** |  | **Rural** |  | **Urban** | | | **Low** | | | **Medium** | | | **High** | | |
|  | **Mean** | **95% CI** | | **Mean** | **95% CI** | | **Mean** | **95% CI** | | **Mean** | **95% CI** | | **Mean** | **95% CI** | |
| **Energy (kcal)** |  |  |  |  |  |  |  |  |  |  |  |  |  |  |  |
| Scenario 1 | 1839.7 | 1757.0 | 1922.3 | 1809.3 | 1726.3 | 1892.4 | 1788.9 | 1691.7 | 1886.2 | 1785.7 | 1692.9 | 1878.5 | 1849.1 | 1742.0 | 1956.2 |
| Scenario 2 | 1678.6 | 1599.6 | 1757.7 | 1586.6 | 1512.5 | 1660.8 | 1643.5 | 1551.5 | 1735.5 | 1596.3 | 1509.5 | 1683.1 | 1602.8 | 1510.2 | 1695.4 |
| Scenario 3 | 1676.1 | 1597.0 | 1755.3 | 1583.1 | 1509.3 | 1657.0 | 1641.6 | 1549.5 | 1733.6 | 1593.1 | 1506.3 | 1680.0 | 1598.9 | 1506.9 | 1691.0 |
| Scenario 4 | 1608.8 | 1531.2 | 1686.4 | 1508.9 | 1435.7 | 1582.2 | 1584.5 | 1492.5 | 1676.4 | 1519.4 | 1433.0 | 1605.7 | 1520.3 | 1430.0 | 1610.6 |
| **Saturated fat (g)** |  |  |  |  |  |  |  |  |  |  |  |  |  |  |  |
| Scenario 1 | 19.2 | 17.8 | 20.5 | 20.8 | 19.5 | 22.2 | 17.7 | 16.3 | 19.2 | 19.7 | 18.4 | 21.1 | 22.1 | 20.3 | 23.8 |
| Scenario 2 | 15.9 | 14.8 | 17.0 | 16.5 | 15.4 | 17.5 | 15.0 | 13.7 | 16.4 | 16.0 | 14.8 | 17.2 | 17.1 | 15.9 | 18.4 |
| Scenario 3 | 15.8 | 14.7 | 16.9 | 16.3 | 15.3 | 17.3 | 14.9 | 13.6 | 16.3 | 15.8 | 14.6 | 17.1 | 17.0 | 15.7 | 18.2 |
| Scenario 4 | 15.4 | 14.3 | 16.5 | 15.8 | 14.7 | 16.8 | 14.6 | 13.3 | 15.9 | 15.3 | 14.1 | 16.5 | 16.4 | 15.1 | 17.6 |
| **Trans fat (g)** |  |  |  |  |  |  |  |  |  |  |  |  |  |  |  |
| Scenario 1 | 0.1 | 0.1 | 0.2 | 0.2 | 0.2 | 0.2 | 0.1 | 0.1 | 0.2 | 0.2 | 0.1 | 0.2 | 0.2 | 0.2 | 0.3 |
| Scenario 2 | 0.1 | 0.1 | 0.1 | 0.1 | 0.1 | 0.2 | 0.1 | 0.1 | 0.1 | 0.1 | 0.1 | 0.1 | 0.1 | 0.1 | 0.2 |
| Scenario 3 | 0.1 | 0.1 | 0.1 | 0.1 | 0.1 | 0.2 | 0.1 | 0.1 | 0.1 | 0.1 | 0.1 | 0.1 | 0.1 | 0.1 | 0.2 |
| Scenario 4 | 0.1 | 0.1 | 0.1 | 0.1 | 0.1 | 0.2 | 0.1 | 0.1 | 0.1 | 0.1 | 0.1 | 0.1 | 0.1 | 0.1 | 0.2 |
| **Added sugars (g)** |  |  |  |  |  |  |  |  |  |  |  |  |  |  |  |
| Scenario 1 | 49.7 | 45.6 | 53.8 | 59.7 | 54.5 | 64.9 | 50.4 | 44.8 | 55.9 | 52.8 | 48.3 | 57.3 | 63.0 | 55.7 | 70.3 |
| Scenario 2 | 25.3 | 22.6 | 28.1 | 28.7 | 24.4 | 33.0 | 26.4 | 22.7 | 30.2 | 25.3 | 22.7 | 27.8 | 30.1 | 23.7 | 36.5 |
| Scenario 3 | 25.3 | 22.6 | 28.1 | 28.7 | 24.4 | 33.0 | 26.4 | 22.7 | 30.2 | 25.3 | 22.7 | 27.8 | 30.1 | 23.7 | 36.5 |
| Scenario 4 | 22.8 | 20.1 | 25.6 | 24.2 | 20.3 | 28.0 | 24.3 | 20.6 | 27.9 | 22.1 | 19.7 | 24.6 | 24.7 | 19.0 | 30.4 |
| **Sodium (mg)** |  |  |  |  |  |  |  |  |  |  |  |  |  |  |  |
| Scenario 1 | 7586.8 | 6623.3 | 8550.3 | 9217.1 | 8207.1 | 10227.1 | 7959.2 | 6496.8 | 9421.5 | 7955.3 | 7115.2 | 8795.5 | 9727.9 | 8362.7 | 11093.1 |
| Scenario 2 | 6654.7 | 5900.3 | 7409.2 | 8043.4 | 7186.0 | 8900.9 | 7146.3 | 5811.1 | 8481.5 | 7127.9 | 6347.1 | 7908.6 | 8300.0 | 7236.5 | 9363.6 |
| Scenario 3 | 6643.5 | 5888.3 | 7398.7 | 8023.0 | 7165.0 | 8880.9 | 7138.1 | 5802.1 | 8474.1 | 7112.5 | 6330.9 | 7894.2 | 8275.6 | 7211.8 | 9339.5 |
| Scenario 4 | 6484.8 | 5732.2 | 7237.3 | 7765.2 | 6904.8 | 8625.5 | 6985.7 | 5652.3 | 8319.1 | 6911.5 | 6125.0 | 7698.1 | 7986.2 | 6917.4 | 9055.0 |

FOPWL: Front-of-Pack Warning Label. Scenario 1: Current intake of Mexican population; Scenario 2: Energy and nutrient intake after the simulation during the 1st phase of the norm; Scenario 3: Energy and nutrient intake after the simulation during the 2nd phase of the norm; Scenario 4: Energy and nutrient intake after the simulation during the 3rd phase of the norm.

**Additional file 1: Supplementary Table 1. Critical nutrient intake before and after the replacement with FOPWL criteria by sociodemographic characteristics. Mexico, ENSANUT 2016.**

|  | **Region** | | | | | | | | | | | |
| --- | --- | --- | --- | --- | --- | --- | --- | --- | --- | --- | --- | --- |
| **Critical Nutrients** | **North** | | | **Center** | | | **South** | | | **Mexico State and Mexico City** | | |
|  | **Mean** | **95% CI** | | **Mean** | **95% CI** | | **Mean** | **95% CI** | | **Mean** | **95% CI** | |
| **Energy (kcal)** |  |  |  |  |  |  |  |  |  |  |  |  |
| Scenario 1 | 1865.9 | 1676.515 | 2055.285 | 1835.0 | 1719.5 | 1950.6 | 1780.0 | 1687.4 | 1872.6 | 1791.1 | 1646.3 | 1935.9 |
| Scenario 2 | 1598.548 | 1414.528 | 1782.568 | 1603.9 | 1508.1 | 1699.8 | 1634.1 | 1551.0 | 1717.2 | 1588.2 | 1454.8 | 1721.6 |
| Scenario 3 | 1594.554 | 1411.313 | 1777.796 | 1600.6 | 1504.7 | 1696.5 | 1631.3 | 1548.9 | 1713.8 | 1585.2 | 1451.6 | 1718.7 |
| Scenario 4 | 1525.682 | 1335.685 | 1715.68 | 1516.7 | 1427.8 | 1605.6 | 1566.0 | 1483.5 | 1648.4 | 1518.0 | 1386.3 | 1649.8 |
| **Saturated fat (g)** |  |  |  |  |  |  |  |  |  |  |  |  |
| Scenario 1 | 21.8 | 19.2 | 24.3 | 21.2 | 19.0 | 23.4 | 18.7 | 17.3 | 20.2 | 20.4 | 18.0 | 22.8 |
| Scenario 2 | 17.4 | 15.1 | 19.8 | 16.3 | 15.0 | 17.6 | 15.7 | 14.6 | 16.8 | 16.3 | 13.9 | 18.7 |
| Scenario 3 | 17.3 | 14.9 | 19.6 | 16.1 | 14.8 | 17.4 | 15.5 | 14.4 | 16.6 | 16.2 | 13.8 | 18.6 |
| Scenario 4 | 16.8 | 14.4 | 19.2 | 15.5 | 14.2 | 16.7 | 15.2 | 14.1 | 16.3 | 15.6 | 13.3 | 17.9 |
| **Trans fat (g)** |  |  |  |  |  |  |  |  |  |  |  |  |
| Scenario 1 | 0.2 | 0.1 | 0.4 | 0.2 | 0.1 | 0.2 | 0.2 | 0.1 | 0.2 | 0.2 | 0.1 | 0.2 |
| Scenario 2 | 0.2 | 0.1 | 0.3 | 0.1 | 0.1 | 0.1 | 0.1 | 0.1 | 0.1 | 0.1 | 0.1 | 0.2 |
| Scenario 3 | 0.2 | 0.1 | 0.3 | 0.1 | 0.1 | 0.1 | 0.1 | 0.1 | 0.1 | 0.1 | 0.1 | 0.2 |
| Scenario 4 | 0.2 | 0.1 | 0.3 | 0.1 | 0.1 | 0.1 | 0.1 | 0.1 | 0.1 | 0.1 | 0.0 | 0.2 |
| **Added sugars (g)** |  |  |  |  |  |  |  |  |  |  |  |  |
| Scenario 1 | 56.2 | 50.1 | 62.4 | 61.1 | 53.2 | 69.1 | 51.0 | 45.8 | 56.3 | 62.1 | 48.3 | 75.9 |
| Scenario 2 | 19.5 | 16.6 | 22.5 | 29.6 | 23.2 | 35.9 | 28.8 | 25.4 | 32.1 | 32.6 | 19.1 | 46.1 |
| Scenario 3 | 19.5 | 16.6 | 22.5 | 29.6 | 23.2 | 35.9 | 28.8 | 25.4 | 32.2 | 32.6 | 19.1 | 46.1 |
| Scenario 4 | 14.6 | 11.2 | 18.0 | 24.5 | 19.8 | 29.3 | 26.4 | 23.3 | 29.5 | 28.8 | 15.7 | 41.8 |
| **Sodium (mg)** |  |  |  |  |  |  |  |  |  |  |  |  |
| Scenario 1 | 9773.0 | 7348.3 | 12197.6 | 9354.0 | 7856.3 | 10851.8 | 6877.7 | 6085.1 | 7670.4 | 10216.5 | 8374.4 | 12058.7 |
| Scenario 2 | 7453.6 | 5972.5 | 8934.7 | 8326.0 | 6961.8 | 9690.1 | 6302.0 | 5528.2 | 7075.7 | 9350.7 | 7594.0 | 11107.5 |
| Scenario 3 | 7429.9 | 5950.4 | 8909.3 | 8309.9 | 6945.4 | 9674.3 | 6286.0 | 5507.4 | 7064.6 | 9330.7 | 7576.0 | 11085.4 |
| Scenario 4 | 7122.0 | 5636.6 | 8607.5 | 8052.8 | 6689.0 | 9416.7 | 6104.6 | 5319.5 | 6889.8 | 9138.8 | 7384.1 | 10893.6 |

FOPWL: Front-of-Pack Warning Label. Scenario 1: Current intake of Mexican population; Scenario 2: Energy and nutrient intake after the simulation during the 1st phase of the norm; Scenario 3: Energy and nutrient intake after the simulation during the 2nd phase of the norm; Scenario 4: Energy and nutrient intake after the simulation during the 3rd phase of the norm.
